# Traumatic Brain Injury in Honduras: The Use of a Paper-based Surveillance System to Characterize Injuries Patterns

**DOI:** 10.5195/ijms.2022.1384

**Published:** 2022-09-16

**Authors:** Erica Johnson, Cristina Rodriguez, Juan C. Puyana, Francisco J. Bonilla-Escobar

**Affiliations:** 1 MD, University of Pittsburgh, Pittsburgh, PA, United States.; 2 MD, Tegucigalpa, Honduras.; 3 MD, FRCSC, FACS, FACCP. School of Medicine, Department of Surgery, Professor of Surgery, Critical Care Medicine, and Clinical Translational Science, Director for Global Health-Surgery, University of Pittsburgh, Pittsburgh, PA, United States. Editorial Board Member, IJMS; 4 MD, MSc, PhD(c). Researcher, Department of Ophthalmology; Institute for Clinical Research Education (ICRE), University of Pittsburgh, Pittsburgh, PA, United States. CEO, Fundación Somos Ciencia al Servicio de la Comunidad, Fundación SCISCO/Science to Serve the Community Foundation, SCISCO Foundation, Cali, Colombia. Grupo de investigación en Visión y Salud Ocular, VISOC, Universidad del Valle, Cali, Colombia. Editor in Chief, IJMS

**Keywords:** Wounds and Injuries, Nervous System Trauma, Trauma Centers, Violence, Honduras, Traumatic Brain Injuries (Source: MeSH-NLM)

## Abstract

**Background:**

Traumatic brain injuries (TBI) are a leading cause of death and disability worldwide. Violence is the leading cause of mortality in Honduras. However, the incidence and impact of TBI in this low-middle income country (LMIC) is unknown. The aim of this study is to describe the epidemiology of TBI in Honduras, as captured by an injury surveillance tool in the country’s major referral center.

**Methods:**

A cross sectional review of all TBI-related emergency department visits at the main referral hospital in Honduras from January to December 2013 was conducted. The calculation of descriptive statistics from Injury Surveillance System (InSS) data was performed.

**Results:**

Of 17,971 total injuries seen in 2013, 20% were traumatic brain injuries (n=3,588). The main mechanisms of injury were falls (41.11%), road traffic accidents (23.91%), blunt trauma (20.82%), penetrating knife injuries (5.85%), and firearm injuries (2.26%). Most TBI were classified as mild; 99.69% (Glasgow Coma Scale=15). Emergency room mortality was low (1.11%). The modified Kampala Trauma Score median was 8 (interquartile range 7–8).

**Conclusion:**

Mild TBI accounts for a significant percentage of all injuries presenting to a high-volume referral center in Honduras in 2013. Despite the high incidence of violence in this country, most TBI were accidental, secondary to road traffic accidents and falls. Further research is required with more recent data as well as with prospective data collection methods.

## Introduction

Traumatic brain injury (TBI) causes death and significant disability worldwide.^1–3^ Estimates of the global incidence of TBI are as high as 69 million per year.^4^ The impact is especially high in low- and middle-income countries, where the population is at risk for injury due to epidemiological and environmental factors, and have three-times the incidence rate of high-income countries. The affected populations are often younger and live below the poverty line.^5^ In addition, most countries in Latin America experience high rates of road traffic collisions and exceedingly high rates of interpersonal violence.^6^

Results from the WHO Global Burden of Disease Study suggests that Latin American countries have the highest incidence of intracranial injury in the world.^7,8^ TBI age-standardized prevalence has increased from 1990 to 2016 by 8.4% and is responsible for 8.1 million years of life lived with disability. The most common causes of TBI have been reported as falls and road injuries.^9^ There is a need of information regarding TBI in Latin American countries to inform public health policies and implement trauma protocols to reduce this burden.^10^

The Central American country of Honduras, a low-middle income country (LMIC), has a high rate of violence.^11^ In 2014, the United Nations ranked Honduras as the world’s most violent country, with homicide rates of 85.5 per 100,000 inhabitants. Males were mainly affected (91.6%) and were most often injured with firearms (83%).^12,13^ Despite the high rate of violence, there are few published studies that have assessed the impact of traumatic injury in Honduras.^2,14^ It is difficult to obtain data on injury patterns in Honduras due to the lack of a formal trauma registry.^15^ Recently, an incidence of 279 TBIs and a prevalence of 567 per 100,000 inhabitants were reported in Honduras, with an increasing trend of 30% in both indicators when comparing the year 1990 to 2016.^9^

The Injury Surveillance System (InSS) is a paper-based injury surveillance system used to capture epidemiological data on injury-related visits to the University’s Medical School Hospital (UMSH) in Tegucigalpa, Honduras. The goal of the InSS is to measure and study injury epidemiology and trauma-related outcomes in the absence of a trauma registry. The InSS was established in 2005 through initiatives and funding from the United States Center for Disease Control (CDC), the Pan-American Health Organization (PAHO), and the United Nations Development Programe (UNDP). Paper-based trauma surveillance systems have been successfully used to collect injury data in emergency departments in other low- and middle-income countries, such as Colombia, El Salvador, Peru, and Jamaica.^16^ The InSS was last validated in 2013 and therefore, well suited for this study. To the best of our knowledge, there is no recent data available from any hospital-based surveillance systems on TBI in the country.

The aim of this research was to use data from the InSS to describe the patients with traumatic brain injuries, the characteristics of the injuries, and the patients’ outcomes in a major referral center in Tegucigalpa, the capital of Honduras. This research could help establish a baseline of TBI in the city, as well as provide more information on patient characteristics towards the promotion of novel assessments of this issue and future improvement of healthcare provision and prevention.

## Methods

A cross-sectional review of all injury-related Emergency Department visits to the University’s Medical School Hospital (UMSH) in Tegucigalpa from January 1st, 2013 to December 31st, 2013 was conducted. The UMSH is the main referral center for 64 primary care health centers and five hospitals in the Central District of Honduras, home to 1.8 million inhabitants. The hospital recorded an estimated 87,000 patients visits a year, with approximately 15% of admissions due to trauma. Injury data from the injury surveillance system (InSS), a paper-based instrument that was first implemented in 2005 to register all injury-related visits to the UMSH emergency room, was obtained. The goal of the InSS is to obtain basic epidemiological information on traumatic injuries.^16–18^

A hospital worker at the UMSH completed an InSS document for every patient that arrived at the Emergency Department by interviewing the patient and/or the family. The paper-based form captures demographic information (age, date of birth, and marital status), descriptions of injury mechanism, injury type and severity, and circumstances of injury. Specific information on injury intentionality, the presence of drug or alcohol use at the time of injury, and on road traffic collisions is included in the form. The InSS also includes some basic outcomes measures, such as the patient’s treatment plan, clinical evaluations, disposition, and mortality.^17^ This information is included in each patient’s chart and later transferred to an electronic record.^18^

A physician evaluated each patient at the emergency room and filled the medical, diagnosis, and treatment sections of the InSS. All patients with TBIs described in the InSS in the diagnosis section of the form were included. TBI was considered as a sudden trauma to the head that could have affected the brain, with different grades of severity from transitory symptoms such as blurry vision, confusion, and loss of consciousness to severe loss of cognitive and motor responses. Exclusion criteria included not having at least 20% of the specifications; however, all prospective subjects met the inclusion criteria and were used in this study.

For the purposes of this study, all InSS data were transferred to Stata 14^®^ (StataCorp, TX, USA) for review and analysis. The quantitative variables were described using central tendency and dispersion measures while categorical variables were described with frequencies and percentages. Chi-squared tests were performed to compare groups for categorical variables. Injury rates per 100,000 inhabitants and 95% exact confidence intervals (95%CI) were calculated based on a binomial distribution (N=8,303,771 inhabitants, National Institute of Statistics of Honduras). The mortality risk was assessed using the modified Kampala Trauma Score, a validated tool to assess injury severity. The modified Kampala Trauma Score, calculated using age, systolic blood pressure, respiratory rate, neurologic status, and number of injuries, is used for risk stratification of patients in resource-limited settings.^22^

Injuries were either classified as intentional or unintentional. Intentional injuries were either violent interpersonal injuries or self-inflicted. Unintentional injuries included both falls and road traffic collisions.

This study was approved by the Institutional Review Board of the University of Pittsburgh (PRO17080111) as a part of a wider study assessing trauma in Honduras.

## Results

In 2013, a total of 17,971 patients were registered in the InSS over the period of January 1st, 2013 to December 31st, 2013, resulting in an injury rate of 216.42 per 100,000 inhabitants (95% CI: 213.27–219.60). There were 3,588 TBIs captured in the InSS, accounting for 19.97% of all injuries over the study period (3,588 /17,971 injuries in 2013, Table 1). Most patients were treated and discharged on the same day of arrival (54.9%, 9,855 patients), and 44.7% of patients were hospitalized (8,021).

**Figure 1.**
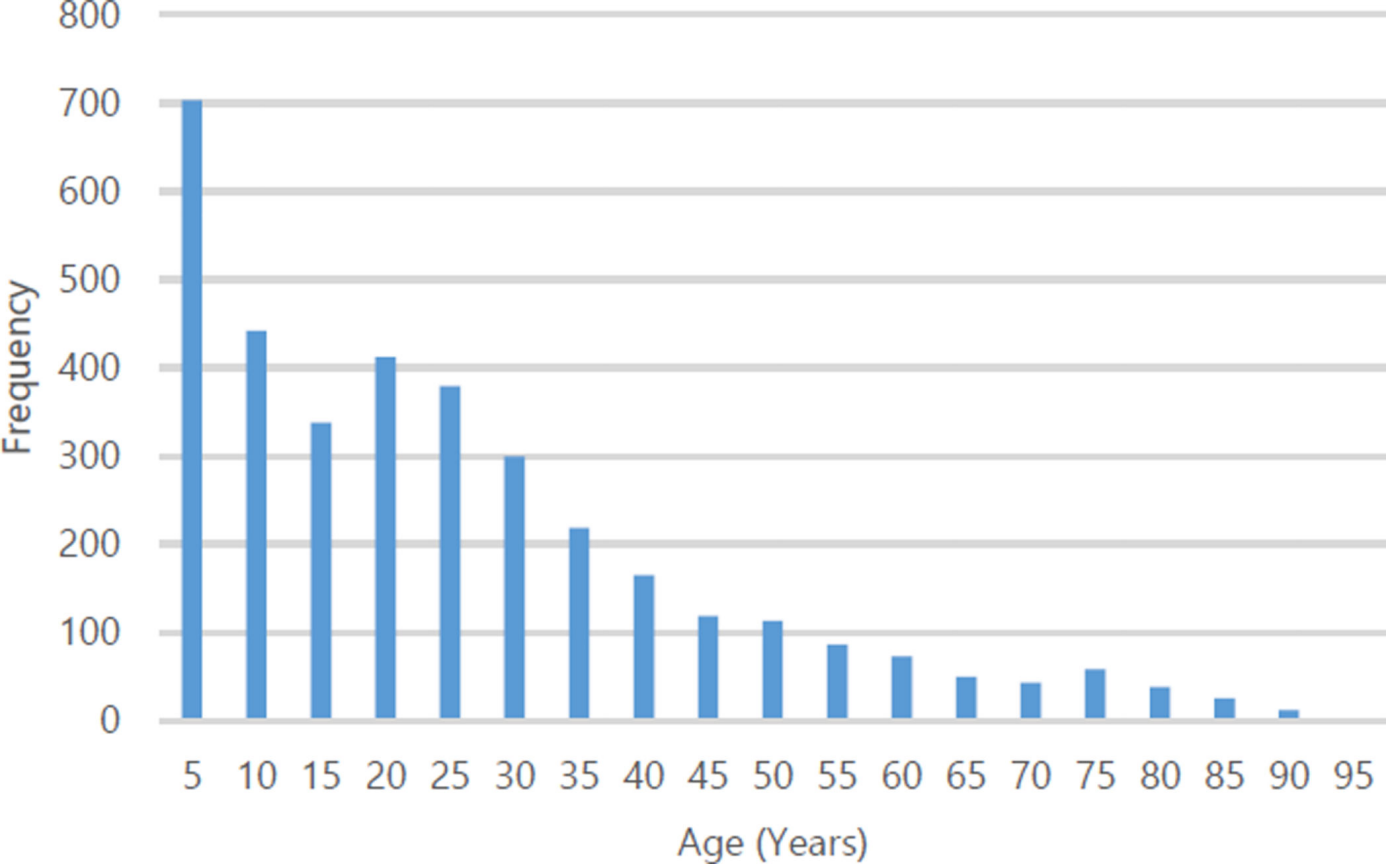
Age Distribution of Patients with Traumatic Brain Injury in Honduras, 2013.

Among the recorded traumatic brain injuries, 14.88% were open injuries. The male-to-female injury rate was 2:1 (Table 2). The average age of injury was 23±19 years, with the majority occurring between 0–17 years of age (46.42%, Figure 1).

**Figure 2.**
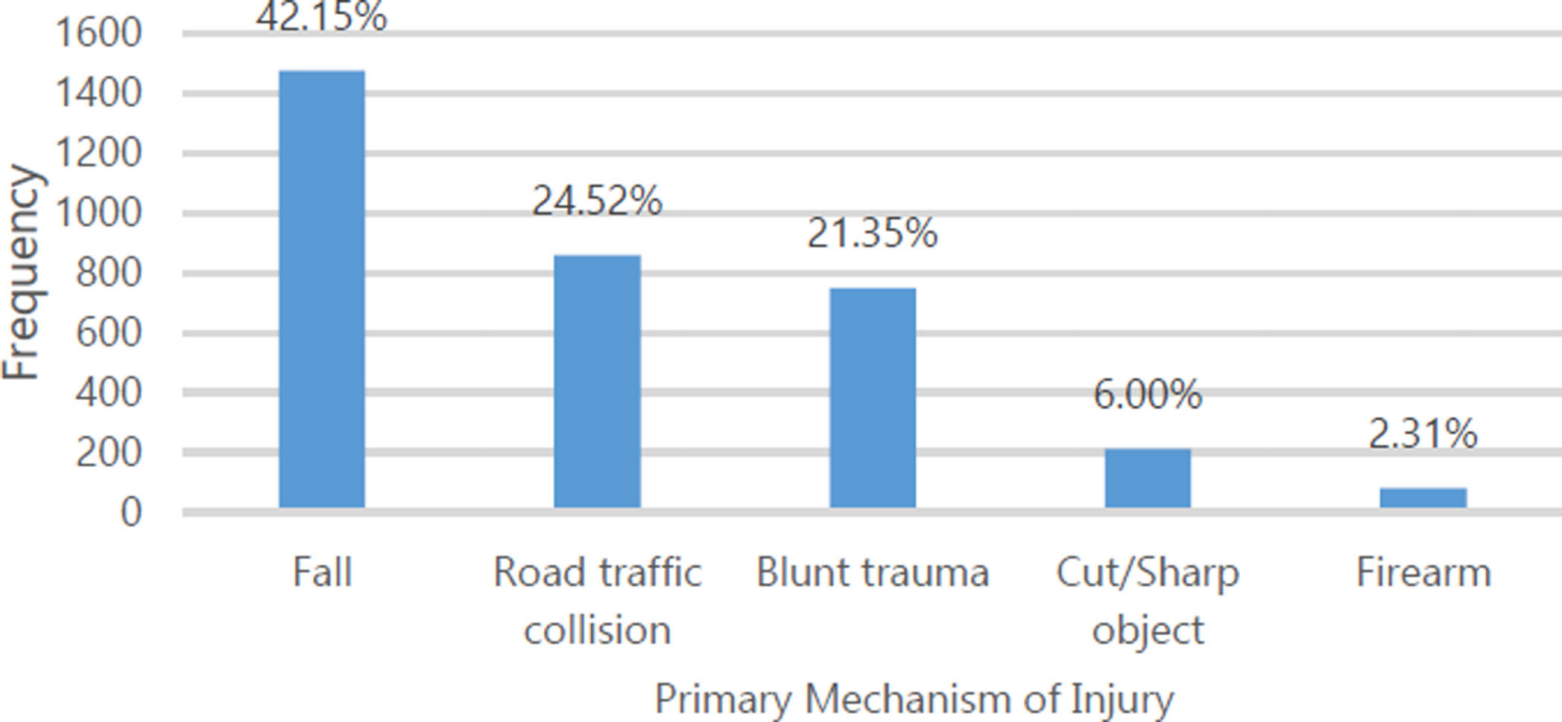
Primary Mechanism of Injury for Patients with Traumatic Injury, 2013.

**Figure 3.**
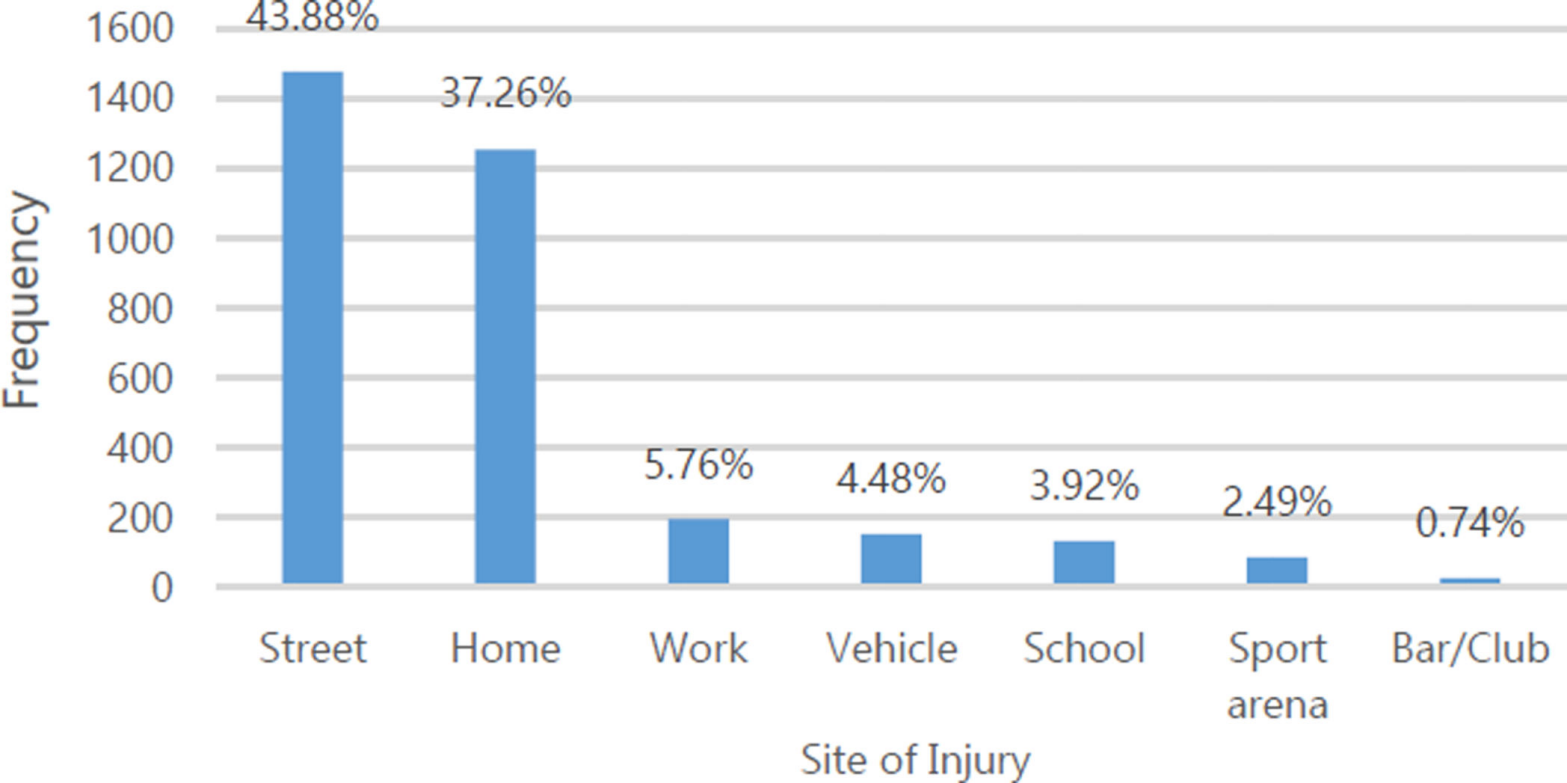
Location at Time of Injury for Patients with Traumatic Brain Injury.

The main mechanisms of injury were falls (41.11%), road traffic (23.91%), and blunt trauma (20.82%, Figure 2). Most injuries occurred at home or in the streets (Figure 3). Overall, the vast majority of injuries were non-intentional (83.39% of patients). Only 16.58% of injuries were intentional, with 16.05% of these due to interpersonal violence and 0.53% due to self-inflected injuries (Table 1). Intentional injuries were more likely among men (p<0.001) and patients aged 18–45 (p<0.001, Table 3).

Most traumatic brain injuries seen at the UMSH were mild. Most patients had a Glasgow Coma Scale (GCS) of 15 on arrival (99.69%). Almost half (43.2%) of the patients were hospitalized, with higher injury rates in males in general (74.7%, p<0.001), individuals aged 0–17 years (51.9%, p<0.001), and more injuries occurring at home or in the streets (33.3% or 48.6%, respectively, p<0.001), caused by falls (40.3%, p<0.001), and during recreational activities or travelling (39.8% or 33.2%, respectively, p<0.001, Table 3).

Only 1.11% of the patients with a TBI died in the emergency room (Table 2). They had multiple traumas including cervical (5%), thoracic (7.5%), abdominal (7.5%), and muscle/bone(15%). These patients also had severe injuries that required surgery in 80% of the cases. Death at emergency room was significant in males (87.5%, p=0.02) and individuals with ages between 18 and 45 years (66.7%, p<0.001), and caused by non-intentional injuries (67.5%, p=0.007), in road traffic collisions (50%, p<0.001), working or travelling (45% and 40%, respectively, p<0.001), and in the streets (73.1%, p=0.005, Table 3). The modified Kampala Trauma Score median was 8 (interquartile range 7–8).

## Discussion

### Traumatic Brain Injury in Honduras

In general, traumatic brain injury accounted for a significant proportion of emergency department visits during the study period. This is similar to incidence rates in other Caribbean countries. An evaluation of Emergency Department admissions in Haiti demonstrated that neurotrauma, including both brain and spinal cord injuries, accounted for 28% of visits.^19^ At UMSH in Honduras, a majority of the injuries were mild, as assessed by the Glasgow Coma Scale, and with a low risk of mortality, as measured by modified Kampala Trauma Score.

The burden of TBI in Honduras is mainly in the youth and children. The average age of patients seen at the UMSH was 23, and the largest proportion of injuries occurred in children aged 0–17. This is in contrast to epidemiological data in the United States and Europe, where both the young and the elderly are affected by TBI. A recent systematic review of the epidemiology of traumatic brain injury in Europe found that TBI showed a bimodal distribution, predominantly affecting those younger than 25 years or older than 75 years.^20^ Males were disproportionately affected by TBI, consistent with existing epidemiological studies of these injuries in low- and middle-income countries. The main mechanisms of injury were falls and road traffic collisions. This data is similar to that obtained from traumatic brain injury emergency department admissions in the United States, where the common causes of TBI were falls (47%), being struck by/against an object (15%), and motor vehicle collisions (14%).^21^ In contrast, Latin America and the Caribbean have higher rates of TBI due to road traffic incidents.^7^

Overall, this study effectively used the InSS data to characterize TBI and potential risk factors for hospitalization, and common mechanisms of injury and outcomes using the best available information on injuries in Honduras. This data could be used to develop targeted measures to inform the development of preventive strategies, optimization of treatment, and reallocation of scarce healthcare resources. For example, public health strategies targeting motor vehicle accidents and falls could decrease the occurrence of TBI in Honduras. The introduction of trauma registry systems has been shown to improve outcomes, and even to decrease mortality.^22^ Data from injury surveillance systems and trauma registries may be used to develop standardized trauma protocols (STP), which have been shown to improve outcomes in traumatic brain injury. One retrospective cohort study investigating the use of an STP at a Level 1 trauma center in Colombia found improved outcomes after STP implementation: in-hospital mortality decreased (p = 0.024) and discharge GCS increased from a median of 10 to a median of 14 (p = 0.034).^23,24^

In addition, improving triage and diverting mild TBI to other facilities could allow a more efficient use of limited resources in managing trauma. In this study, most TBI seen at the UMSH were mild with a low risk of mortality; these injuries could potentially be managed at other facilities, freeing resources for the management of higher acuity trauma.

### Limitations

There are several limitations of this study. Firstly, the InSS only captures injuries in those stable enough for emergency department admission or transfer, thus providing a limited view of overall injury patterns. However, in the absence of a codified trauma registry, the InSS does provide the best available data for characterizing injuries in Honduras. Secondly, the use of a paper-based system could also contribute to variability and error in the way injury data is recorded and coded. We did seek to minimize this error by providing clear instructions on the use of the InSS to those recording this data. Additional studies are necessary to determine the nature of these findings. Finally, the InSS does not describe injuries based on the International Classification of Disease (ICD) coding system, making it difficult to categorize Honduras’ injuries and make comparisons with other countries. However, the InSS contains sufficient data to record a Kampala Trauma Score, a measure used in resource limited settings to characterize injury severity. Finally, the last validation of the InSS was conducted in 2013, therefore, this is the last available data for research that we had access to. A further research updating our data as well as with prospective data collection methods could provide a bigger picture of the situation towards preventive strategies.

### Conclusion

The paper-based Injury Surveillance System provided sufficient data on traumatic brain injury in Honduras to characterize risk factors, mechanism of injury, and injury severity. Trauma registries provide an important tool to improve understanding of epidemiology of injury, treatment regimens, and practice patterns in LIMCs. Trauma surveillance systems have the potential to transform trauma care in LMICs by identifying the specific challenges and opportunities unique to the region, as evidenced by registries instituted in Paraguay, Jamaica and Cali-Colombia.^16^ The InSS provides an important first step in characterizing injury patterns in Honduras, and further research in injuries is required to promote better patient-care and trauma surveillance.

## Figures and Tables

**Table 1.** Characteristics of Traumatic Brain Injuries in Honduras, 2013.

Factor	Frequency (n=3588)
Sex, n (%)	
Female	1028 (28.7%)
Male	2554 (71.3%)
Age, median (IQR)	19 (7 – 33)
Age categories in years, n (%)	
0–17	1659 (46.4%)
18–45	1415 (39.6%)
46–65	322 (9.0%)
66–95	178 (5.0%)
Pregnancy, n (%)	12(1.2%)
TBI severity based on Glasgow coma scale, n (%)	
Mild	3582 (99.9%)
Moderate	3 (0.1%)
Severe	2 (0.1%)
Other affected anatomical sites, n (%)	
Eyes	46(1.3%)
Nose	28 (0.8%)
Neck	22 (0.6%)
Others	69(1.9%)
Place of injury, n (%)	
Street	1478 (43.9%)
Home	1255 (37.3%)
Workplace	194 (5.8%)
Vehicle	151 (4.5%)
School	132 (3.9%)
Sport arena	84 (2.5%)
Other	74 (2.1%)
Main mechanism of injury, n (%)	
Fall	1475 (42.2%)
Road traffic collision	858 (24.5%)
Blunt force	747 (21.3%)
Sharpe object injury	210 (6.0%)
Gunshot	81 (2.3%)
Other	128 (3.6%)
Activity, n (%)	
Recreation	1302 (40.2%)
Traveling	1007 (31.1%)
Working	472 (13.2%)
Drinking alcohol	152 (4.7%)
Working/cleaning the house	110(3.4%)
Practicing sport	106 (3.3%)
Other	86 (2.4%)
Intentionality	
Unintentional	2991 (83.4%)
Intentional	576 (16.1%)
Self-inflicted	20 (0.6%)
Outcome at Emergency	
Discharged	1997 (55.7%)
Hospitalized	1549 (43.2%)
Dead	40(1.1%)

SD: Standard deviation.

ɸ GCS is categorized as mild (13–15), moderate (9–12) and severe (≤ 8).

¥ Calculated only for women (n=1,028).

**Table 2.** Patients with Traumatic Brain Injury Characteristics by Intention of the Injury in Honduras.

Characteristics	Intentional TBI (n=576)	Unintentional TBI (n=3,012)	p-value
Sex*			p<0.001
Male	485	2,069	
Female	91	937	
Age*			p<0.001
0–17 years, n	96	1,563	
18–45 years, n	396	1,019	
46–98 years, n	82	418	
Pregnancy ^ɸ^			p=0.291
Pregnant	2	10	
Not pregnant	90	929	
Glasgow Coma Scale^¥^			p=0.583
Mild, n	575	3,008	
Moderate, n	0	2	
Severe, n	1	2	

* Significant at p=0.05.

ɸ Calculated only for women (n=1,028).

¥ GCS is categorized as mild (13–15), moderate (9–12) and severe (< 8)

**Table 3.** Patients with Traumatic Brain Injuries Characteristics Based on Status at Discharge (Hospitalization or Death) from the Emergency Room.

Characteristics	Status			Hospitalized		
	Alive (n=3,548)	Died (n=40)	p-value	No (n=2,039)	Yes (n = 1,549)	p-value
Sex, n (%)			0.023			<0.001
Female	1,023 (28.9%)	5 (12.5%)		636 (31.3%)	392 (25.3%)	
Male	2,519 (71.1%)	35 (87.5%)		1,398 (68.7%)	1,156 (74.7%)	
Age, median (IQR)	19 (7–33)	27 (20–44)	<0.001	21 (7–34)	17 (8–31)	0.006
Age groups, n (%)			<0.001			<0.001
0–17	1,654 (46.8%)	5 (12.8%)		859 (42.2%)	800 (51.9%)	
18–45	1,389 (39.3%)	26 (66.7%)		863 (42.4%)	552 (35.8%)	
46–65	320 (9.1%)	2 (5.1%)		199 (9.8%)	123 (8.0%)	
66–95	172 (4.9%)	6 (15.4%)		113 (5.6%)	65 (4.2%)	
Place of injury, n (%)			0.005			<0.001
Home	1,253 (37.5%)	2 (7.7%)		781 (40.2%)	474 (33.3%)	
School	132 (3.9%)	0 (0.0%)		84 (4.3%)	48 (3.4%)	
Street	1,459 (43.7%)	19 (73.1%)		787 (40.5%)	691 (48.6%)	
Workplace	192 (5.7%)	2 (7.7%)		114(5.9%)	80 (5.6%)	
Vehicle	148 (4.4%)	3 (11.5%)		68 (3.5%)	83 (5.8%)	
Other	158 (4.7%)	0 (0.0%)		111 (5.7%)	47 (3.3%)	
Main Mechanism of Injury, n (%)			<0.001			<0.001
Road traffic collision	839 (24.2%)	19 (50.0%)		368 (18.4%)	490 (32.7%)	
Fall	1,469 (42.4%)	6 (15.8%)		872 (43.6%)	603 (40.3%)	
Blunt force	744 (21.5%)	3 (7.9%)		570 (28.5%)	177 (11.8%)	
Sharpe object injury	208 (6.0%)	2 (5.3%)		108 (5.4%)	102 (6.8%)	
Gunshot	73 (2.1%)	8 (21.1%)		29 (1.4%)	52 (3.5%)	
Other	128 (3.7%)	0 (0.0%)		54 (2.7%)	74 (4.9%)	
Activity, n (%)			<0.001			<0.001
Working	463 (14.4%)	9 (45.0%)		253 (13.3%)	219 (16.4%)	
Recreation	1,301 (40.5%)	1 (5.0%)		771 (40.6%)	531 (39.8%)	
Traveling	999 (31.1%)	8 (40.0%)		564 (29.7%)	443 (33.2%)	
Drinking alcohol	151 (4.7%)	1 (5.0%)		90 (4.7%)	62 (4.6%)	
Other	301 (9.4%)	1 (5.0%)		222 (11.7%)	80 (6.0%)	
Intentionality			0.007			0.06
No-intentional	2,964 (83.6%)	27 (67.5%)		1,721 (84.4%)	1,270 (82.0%)	
Intentional	583 (16.4%)	13 (32.5%)		318 (15.6%)	278 (18.0%)	
